# Shared Access to Patient Portals for Older Adults: Implications for Privacy and Digital Health Equity

**DOI:** 10.2196/34628

**Published:** 2022-05-04

**Authors:** Jennifer L Wolff, Vadim Dukhanin, Julia G Burgdorf, Catherine M DesRoches

**Affiliations:** 1 Johns Hopkins Bloomberg School of Public Health Johns Hopkins University Baltimore, MD United States; 2 Center for Home Care Policy & Research Visiting Nurse Service of New York New York, NY United States; 3 OpenNotes Beth Israel Deaconess Medical Center Boston, MA United States

**Keywords:** patient portal, electronic health record, care partners, proxy, health equity, health informatics, health services, elderly, older adults, aging, cognition, health system, care delivery, elderly care

## Abstract

Growing reliance on the patient portal as a mainstream modality in health system interactions necessitates prioritizing digital health equity through systems-level strategies that acknowledge and support all persons. Older adults with physical, cognitive, sensory, and socioeconomic vulnerabilities often rely on the involvement of family and friends in managing their health, but the role of these care partners in health information technology is largely undefined and poorly understood. This viewpoint article discusses challenges and opportunities of systematic engagement of care partners through shared access to the patient portal that have been amplified in the context of the COVID-19 outbreak and recent implementation of federal information blocking rules to promote information transparency alongside broader shifts toward care delivery innovation and population aging. We describe implementation considerations and the promise of granular, role-based privacy controls in addressing the nuanced and dynamic nature of individual information sharing preferences and fostering person- and family-centered care delivery.

## Introduction

Shifts toward virtual care delivery in response to the COVID-19 pandemic have demonstrated both the promise and difficulty of electronic modalities in reaching patients who are more vulnerable. The patient portal has had a prominent role throughout the pandemic due to its use in telehealth, the scheduling and provision of COVID-19 test results, and more recently the coordination of hospital-based vaccination efforts. Older adults are more commonly affected by physical, cognitive, sensory, and socioeconomic vulnerabilities that amplify the importance of transparent information exchange. Older adults are also highly diverse with respect to technology access and digital health literacy, which affect ease of portal use [1,2]. As the patient portal becomes a mainstream modality in health system interactions, efforts to achieve digital health equity and respect for older adults’ wide-ranging circumstances, preferences, and capabilities must be prioritized.

Organizational efforts to promote use of the patient portal have primarily focused on increasing patient engagement through public awareness campaigns, clinician and staff training, work process redesign, and information technology support [3]. However, millions of older Americans manage their health with the involvement of family, friends, caregivers, and other care partners who are not part of the formal care delivery system [4]. Care partner engagement has a profound effect on patient quality of life, quality of care, and resource use, but is not well supported in care delivery [4,5]. At a basic level, care partners are often unable to access information about patient health and treatments—information that is necessary and appropriate when coordinating or enacting the patient’s care plan. Attaining the full promise of consumer health information technologies will require meeting the needs and preferences of *all* patients, including those who delegate or comanage their care. This viewpoint seeks to raise awareness of the challenges and opportunities of systematic engagement of care partners through shared access to the patient portal, and to highlight policy and practice considerations that affect efforts to expand shared access.

## Shared Portal Access: The Current Landscape

Health systems commonly allow patients to authorize a care partner to “share access” to their portal account in a registration process through which the care partner is granted their own identity credentials (login and password) [6]. Shared (proxy) portal access is thus an existing functionality that respects patient preferences for involving other individuals in their care. Whether care partners access the portal through *shared* access, using their own identity credentials, or *patient* access, using patient identity credentials, has important ramifications for patients, care partners, and clinicians (Table 1). Care partners’ informal use of patient identity credentials obscures whether and when they are involved in electronic interactions. In contrast, shared portal access affords patients greater control over both granting and revoking access to their portal account, and clinicians the ability to identify with whom they are communicating via electronic interactions. The growing capacity of electronic health records to accept patient-generated health information such as patient-reported questionnaires and patient-uploaded legal documents amplifies the importance of using proper identity credentials to the integrity of electronic health information. Shared portal access also confers advantages to care partners, including greater legitimacy in their interactions with health systems, access to timely and comprehensive information about patient health, and a mechanism to interact with clinicians and manage care tasks electronically.

**Table 1:** Care partners and patient portals: implications of using shared (proxy) versus patient access.

Effects	Benefits of care partners’ use of their own identity credentials through shared (proxy) access	Drawbacks of care partners’ use of patients’ identity credentials through patient access
Patient autonomy and control	Patients clarify which care partners they would like to share access to their portal account and retain the ability to revoke access.	Patients share their own identity credentials with care partners, who are not distinguishable from one another or the patient.
Care partner legitimacy	Clinicians are able to discern which care partner they are communicating with electronically when someone other than the patient contacts them.	Clinicians are not able to distinguish between the patient and care partners in electronic interactions and direct messaging.
Transparency and efficiency of triadic interactions with patients and care partners	Clinicians asynchronously interact with the patient and their care partners, facilitating consistent, transparent, and timely information exchange.	Clinicians may not be as direct and honest in their visit notes and direct messages if they are unsure of who is accessing and acting on the information. Inefficiencies may result from coordinating clinician–care partner interactions by telephone.
Integrity of patient-generated information in their health record	Care delivery systems can identify who is responding to portal surveys or uploading legal documents if someone other than the patient.	Care delivery systems cannot discern when care partners respond to patient assessments or upload legal documents, such as advance directives.
Care partner assessment	Clinicians and care delivery systems may field electronic screening assessments of care partners to identify and monitor their capacity and needs.	Clinicians and care delivery systems may not know whether a care partner is involved or which care partner to screen or monitor. Screening assessments must be completed by phone or paper survey.
Tailored support of the care partner	Gathering care partner–reported information enables tailored delivery of education and support to care partners.	Clinicians and care delivery systems may not know when care partners are at risk of burnout or lacking knowledge of patient health and treatments.

A small but growing body of evidence finds that care partners’ registration and use of the patient portal may yield benefit across dimensions of patient and care partner engagement, satisfaction with communication, and confidence managing care [7,8]. Although shared portal access is reportedly desired by patients and valued by families [2,9-11], uptake has been limited [12-14]. Studies involving convenience samples from care delivery organizations indicate that when care partners do access the portal, it is most often informally, using patient identity credentials [13-17]. In a recent study involving a text analysis of 3000 adult portal messages, care partners who direct messaged clinicians with patient credentials identified themselves about half of the time [17].

The reasons for this low uptake are complex and likely multifactorial. Health care organizations may be reluctant to encourage shared access due to misplaced concerns about the privacy requirements of the Health Insurance Portability and Accountability Act [18,19]. Portal design is often not simple or user-friendly [20]. Finally, while federal programs offering incentive payments for electronic health record adoption did require organizations to offer a patient portal, they set a low threshold for the proportion of patients using them, providing little incentive for robust implementation efforts [21].

Little attention has been directed toward identifying organizational best practices for the implementation of shared portal access functionality; where offered, awareness is low and the registration process is cumbersome and not well understood [11,15,22]. Safeguarding the privacy of electronic personal health information is a critical concern to health care systems. However, concern for data privacy may inhibit appropriate and beneficial access to information needed by care partners who are involved in oversight of or delivering hands-on care [23-26]. Provider policies and procedures were cited as a barrier to technology use by nearly half (48.6%) of out-of-home family caregivers in one study [22]. Although decisions about the types of information made available to care partners through the patient portal are made at the organizational level, the heterogeneity and fluidity of older adults’ circumstances and information sharing preferences preclude a uniform, “one size fits all” approach. Access policies must be flexible to support privacy preferences that may shift over time in the context of age-related changes in function [27]. Further, developing strategies that recognize and address older adults’ highly varied circumstances and preferences, such as the involvement of direct care workers when appropriate and desired, will be especially critical if digital health equity is to be achieved [27].

Importantly, decisions about the types of information and functionalities available to care partners made at the organizational level rather than by individual patients can inhibit patient autonomy and care partner access to information, limit clinician insight regarding patient privacy preferences, and reduce the relative advantage of differentiated patient and care partner identity credentials. A historical health record can contain years of data, some of which may be sensitive, such as mental health treatment and diagnoses and information about stigmatized conditions. A patient may have current health issues that they are not ready to share with a care partner. However, that same patient may want their care partner to be able to communicate with a physician or request appointments or prescription refills, but not see their clinical notes. Alternatively, other patients, such as those with memory issues or vision loss, for example, may want a care partner to have access to clinical notes so that they may know what happened during a clinical visit and understand the care plan. No blanket policy for shared access can adequately address these different scenarios.

## A Look Forward

Achieving widespread patient engagement through the patient portal will require that organizations address the diverse needs and preferences of all patients, including those with greater socioeconomic and physical vulnerability [2,28,29]. The current landscape of shared access indicates that multifaceted efforts will be needed to increase awareness, clarify the value and importance of differentiating patient and care partner identity credentials, and simplify registration processes. Online electronic identity proofing, already available at some health systems [30], holds promise for overcoming cumbersome in-person registration processes and paper-based documentation but disproportionately benefits subpopulations with digital health literacy skills and technology access [31]. A recent review of best practices to engage patients in electronic health records recommended that organizational awareness and marketing efforts target high-cost, high-need subpopulations with greater vulnerability [28]. Such efforts should encompass both patients and their care partners, in recognition of the broader social context in which many older adults comanage or delegate care [29,32], and the arbitrary distinction between patient and care partner roles, which may co-occur simultaneously [32]. Organizational efforts to engage care partners must also resonate with the needs and concerns of clinicians and staff by enhancing knowledge about the importance of proper identity credentials and addressing concerns regarding potential impacts on workflows, time demands, or both [2,3,33,34].

Recognizing that registration and use of the patient portal are separate and significant dimensions of patient engagement [2], additional changes will be needed to enhance the usability of the portal among older adults and care partners with less technology “readiness” and experience. Strong evidence finds that simplifying the user interface, reducing technical language, and enhancing the visual layout of content increases the perceived value of the portal by both patients and care partners [29,35]. The development and implementation of electronic health record certification criteria that require vendors to develop granular, role-based privacy controls would be transformative in acknowledging the nuanced, complex, dynamic nature of individuals’ preferences for sharing their health information [27] and affording patients greater control over who has privileges to undertake health management tasks on their behalf [36]. Putting these privacy controls into the hands of patients may provide peace of mind to clinicians concerned about the privacy of their patients’ protected health information.

Benefits of the patient portal have been generally conceptualized as accruing to patients [37]. However, portal benefits including convenience, continuity, activation, and understanding are equally relevant to care partners, who may additionally benefit from greater legitimacy in their interactions with clinicians and staff due to having their own unique identity credentials. Routine assessment and support of family caregivers are elements of high-quality clinical care and robust systems of long-term services and supports but systems-level approaches are lacking and most interventions have been trialed outside care delivery [4,38]. As an existing mechanism to facilitate bidirectional communication and outreach, shared portal access is a relevant tool in efforts to promote a more optimal person- and family-oriented care delivery system [4,39] and address an identified challenge to disseminate novel technologies to support care partners in real-world practice settings [34,40].

## Conclusion

The development and spread of strategies to engage care partners through the patient portal is especially timely. As of April 5, 2021, federal information blocking rules require that health care providers give patients electronic access without charge or delay to all the health information in their electronic medical records through patient portals or third-party smartphone apps, dramatically expanding the comprehensiveness and timeliness of health care information that is available through the patient portal [41]. The importance of transparent processes to systematically normalize the engagement of care partners in electronic interactions will undoubtedly grow in the coming years given increasing reliance on telehealth and electronic information exchange, the growth of patient-generated health data, and the combination of population aging alongside growth in community-based care settings. Most importantly, through clarifying and respecting differentiated identity credentials, shared portal access sets the stage for protecting the privacy and security of personal health information, while supporting a culture of trust, individual rights, and appreciation for the reality of the broader social context in which individuals commonly comanage their care.
